# Urinary exosome proteins PAK6 and EGFR as noninvasive diagnostic biomarkers of diabetic nephropathy

**DOI:** 10.1186/s12882-023-03343-7

**Published:** 2023-10-03

**Authors:** Tao Li, Tian ci Liu, Na Liu, Meng jie Li, Man Zhang

**Affiliations:** 1 Clinical Laboratory Medicine, Beijing Shijitan Hospital, Capital Medical University, Beijing, 100038 China; 2 Beijing Key Laboratory of Urinary Cellular Molecular Diagnostics, Beijing, 100038 China

**Keywords:** Diabetic nephropathy, Urinary exosomes, PAK6, EGFR, Biomarkers

## Abstract

**Objective:**

The actin cytoskeleton plays an essential role in maintaining podocyte functions. However, whether the urinary exosome proteins related to the regulation of the actin cytoskeleton are changed in diabetic nephropathy (DN) is still unknown. This study was to investigate the possibility that related proteins can be applied as diagnostic biomarkers for DN.

**Methods:**

Urinary exosomes were obtained from 144 participants (Discovery phase: n = 72; Validation phase: n = 72) by size exclusion chromatography methods. Proteomic analysis of urinary exosome by LC-MS/MS. Western blot and ELISA were applied to validate the selected urinary exosome proteins. The clinical value of selected urinary exosome proteins was evaluated using correlation and receiver operating characteristic curve analyses.

**Results:**

Fifteen urinary proteins related to the regulation of the actin cytoskeleton were identified in urinary exosomes. Three upregulated proteins were selected, including Serine/threonine-protein kinase PAK6 (PAK6), Epidermal growth factor receptor (EGFR), and SHC-transforming protein 1(SHC1). The expression level of PAK6 and EGFR was negatively correlated with estimated glomerular filtration rate and positively correlated with serum creatinine levels. For diagnosing DN in the discovery phase: the area under curve (AUC) of PAK6 was 0.903, EGFR was 0.842, and the combination of two proteins was 0.912. These better performances were also observed in the validation phase (For PAK6: AUC = 0.829; For EGFR: AUC = 0.797; For PAK6 + EGFR: AUC = 0.897).

**Conclusions:**

Urinary exosome proteins PAK6 and EGFR may be promising and noninvasive biomarkers for diagnosing DN.

**Supplementary Information:**

The online version contains supplementary material available at 10.1186/s12882-023-03343-7.

## Introduction

Diabetic nephropathy (DN) is one of the most major microvascular complications of diabetes, and the major etiology of end-stage renal disease in the world [1]. The pathogenesis of DN is complicated and driven by a variety of factor. Podocytes are critical components of the glomerular filtration barrier. Increasing evidence suggested that various damages under diabetic situations can trigger injury to podocytes, which leads to the effacement of the foot process and apoptosis [2, 3]. These adverse events contribute to the breakdown of the glomerular filtration barrier, and consequently to proteinuria. Sanja et al. proved that the actin cytoskeleton plays an essential role in maintaining functional podocyte structure based on animal models and podocyte cell culture [4]. Studies also pointed out that actin binding and regulatory proteins are essential components of signaling and actin dynamics at focal adhesions in podocytes [5, 6]. Furthermore, lei’s team demonstrated that elevated AEP in podocytes during DN progression and through cleaving cofilin-1 maintains podocyte cytoskeleton dynamics [7]. The common link between proteinuria and podocyte injuries is the actin cytoskeleton, prompting us to spend more time on finding some noninvasive biomarkers for early prediction of DN.

Currently, urine is the second most commonly used biological liquid in clinical diagnosis. Urine commonly contains some epithelial and blood cells, bacteria, viruses and important exosomes [8, 9]. Since the detection of urine exosomes, studies demonstrated that urinary exosomes could serve as novel biomarkers reflecting the physiological and pathophysiological state of the human body [10, 11]. Furthermore, a recent study suggested that urinary exosomes are engaged in the pathophysiological events related to DN, which could potentially provide promising biomarkers and specific therapeutic targets for DN [12]. In a word, urinary exosomes are characterized by a variety of features, including cargo transfer to specific target cells, regulation of intercellular crosstalk, and alteration of the biofunction of recipient cells, which manifest important consequences for the pathogenesis, diagnosis, and treatment of DN [13]. However, the changes in the regulating actin cytoskeleton-related proteins in urinary exosomes are still unknown.

The present study was statistically analyzed on the basis of the quantitative values of urinary exosome proteins associated with the regulation of the actin cytoskeleton. The expression changes of regulating actin cytoskeleton proteins were explored, and further evaluated their correlation with serum biomarkers of kidney function. Finally, the diagnostic efficacy of target urinary proteins was evaluated, which may provide non-invasive, reliable, and specific urinary exosome biomarkers for T2DM patients with kidney injuries.

## Study cohorts and methods

### Study cohorts

**Fig. 1:**
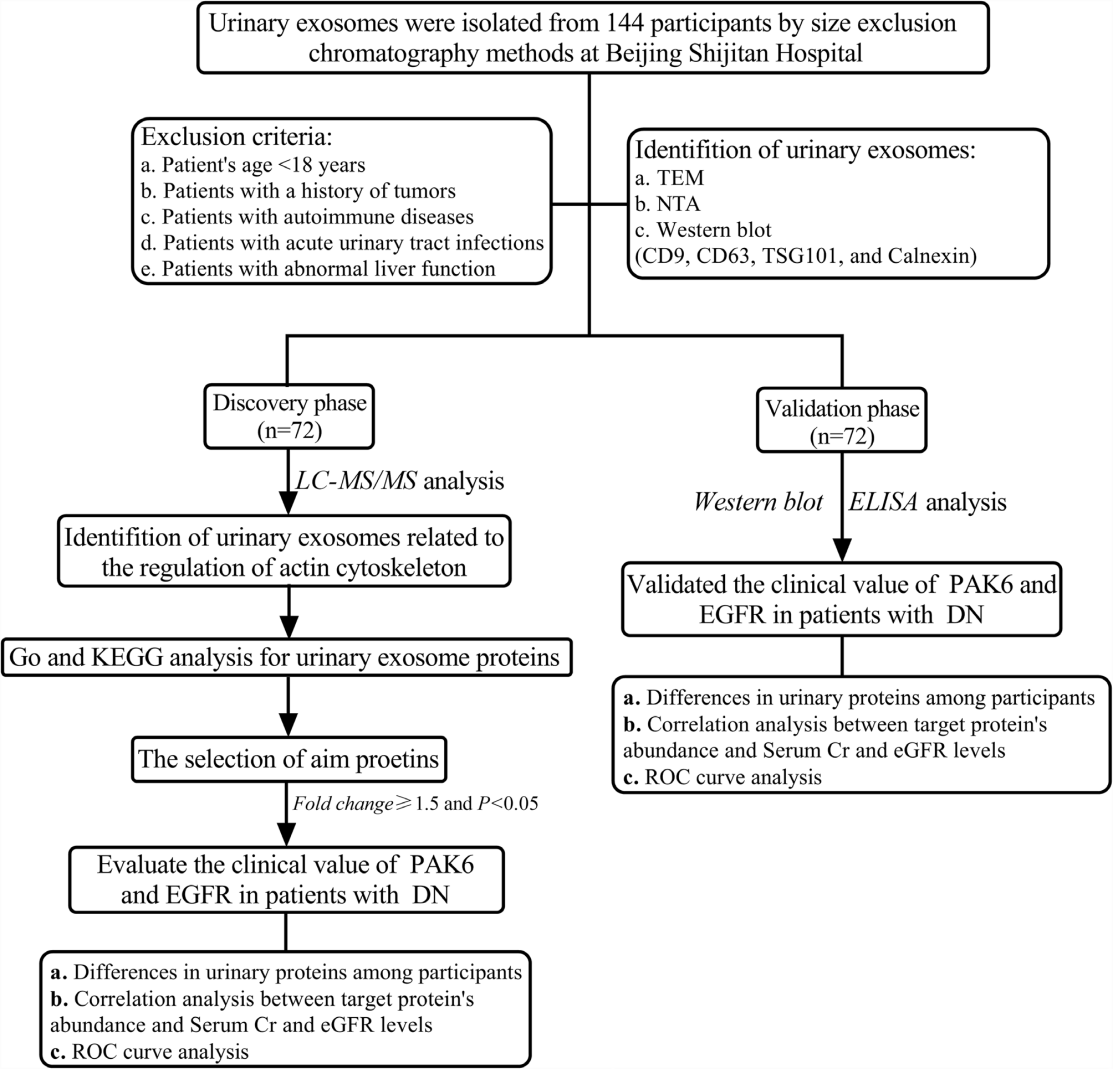
The detailed workflow of this study

From November 2021 to November 2022, forty-eight patients were diagnosed with type 2 diabetes mellitus (T2DM), 48 patients were diagnosed with DN, and 48 healthy subjects were enrolled at Beijing Shijitan Hospital, Capital Medical University. According to the guidelines of American Diabetes Association, T2DM was diagnosed based on the level of fasting blood glucose (FBG) over 7.0 mmol/L, the level of glycosylated hemoglobin (HbA1c) over 6.5%, or the results of blood glucose based on OGTT test (2-hour) over 11.1 mmol/L [14]. The definition of declined estimated glomerular filtration rate (eGFR) was less than 60 mL/min/1.73 m^2^. The definition of albuminuria was the results of urinary albumin/creatinine ratio (UACR) over 30 mg/g. DN was diagnosed clinically by detecting albuminuria and/or declined eGFR without other primary causes of kidney injuries [15]. Patients with aged under 18 years, a history of cancer, chronic liver disease, autoimmune diseases, acute urinary tract infections, or abnormal liver function were excluded. The inclusion criteria for the people selected as a healthy control group were as follows: 1. medical examination results demonstrated that healthy subjects had no cancer, diabetes, heart failure, and chronic liver diseases; 2. healthy people have no history of tumors, chronic liver disease, and chronic renal diseases; 3. all laboratory test results were under normal range; 4. urinary routine test results showed that healthy people without acute urinary tract infections. Clinical features were obtained using electronic medical records at our hospital, for example, height, weight, blood pressure, history of drinking, history of smoking, and history of diabetic family et al. The definition of hypertension was the level of systolic blood pressure (SBP) over 140 mm Hg and/or the level of diastolic blood pressure (DBP) over 90 mm Hg and/or diagnosed by a physician and currently receiving anti-hypertension treatment. The definition of drinking history was a person who was drinking at least once a month on average based on the World Health Organization 2000 guideline [16]. Smoking history was characterized as people who has smoked continuously or cumulatively for 6 months or more in their lifetime [17]. The study was conducted in compliance with the declaration of Helsinki principles and followed by the recommendations of Medical Ethics Committee of Beijing Shijitan Hospital, No. sjtkyll-1x-2021(115). Informed consent was obtained from all subjects enrolled in the present study. This study was approved by the Ethics Committee of Beijing Shijitan Hospital, No. sjtkyll-1x-2021(115). The detailed procedure of the present study was shown in Fig. 1.

### The results of laboratory serum and urinary biomarkers

This study analyzed the serum and urinary biomarkers, which included aspartate aminotransferase (AST), fasting blood glucose (FBG), alanine aminotransferase (ALT), glycated albumin (GA), albumin (ALB), HbA1c, and serum creatinine (Cr) in our laboratory. The Chronic Kidney Disease Epidemiology Collaboration equation was used to evaluate the eGFR levels. UACR was tested through collecting random spot urinary samples for three times. Three UACR tests with at least two positive results.

### Isolation of urinary exosomes

A total of Fifty ml of mid-morning urine sample were obtained from each participant. Collected specimens underwent centrifugation at 1500 g for 10 minutes at a temperature of 4 °C and followed by 10,000 g for 30 minutes to remove the cells and debris. The urine sample was further concentrated to 10 ml using ultrafiltration tubes (Millipore, 10kd). a total of 50 mL of phosphate buffer solution (1xPBS) was utilized to wash the 10/35-nm qEV10 size-exclusion chromatography columns (SECs, H-wayen Biotechnologies, Shang Hai, China). Then, concentrated samples were dropped to SECs. The beginning 10 ml of liquids was discarded, and then 20 ml liquids were saved. Lastly, saved 20 ml liquids were further concentrated to 1 ml using ultrafiltration tubes (Millipore, 10kd) at a speed of 5000 g for 15 ~ 28 minutes and stored at a temperature of -80 °C until utilization.

### The measurement of Transmission Electron microscope (TEM) and nanoparticle tracking analysis (NTA)

#### TEM

Firstly, place 5 µl of each sample onto a clean copper grid. Incubation at a temperature of 26 °C for 5 minutes. Next, extra liquids were absorbed using blotting paper. Secondly a drop of 2% uranyl acetate was added on the exosome sample, and then incubated at a temperature of 26 °C for 1 minute. The surface liquids from one side was absorbed using blotting paper. Finally, the morphology of the urinary exosomes was viewed under the microscope after a 20-minutes drying process (Tecnai G2 Spirit BioTwin, FEI).

#### NTA

Urinary exosome samples were diluted to the suitable concentrations with pre-chilled 1× PBS and used directly for NTA (Zeta View S/N 17–310, PARTICLE METRIX) to detect particle size. The typical result of urinary exosomes concentrations was shown in Supplementary materials 1.

### Mass spectrometry (MS) analysis of urinary exosomes

A simplified procedure for analyzing mass spectrometry is as follows: mobile phase solution A (100% MS water, 0.1% formic acid) and solution B (100% acetonitrile, 0.1% formic acid) were prepared. Peptides were separated using a linear gradient elution method in an analytical column. Data-independent acquisition (DIA) model was utilized to analyze the mass spectra using a QExactive HF-X mass spectrometer (Thermo Fisher). The target value for automatic gain control was 2 × 10^5^, utilizing the NanosprayFlex™ (ESI) ion source, with the ion spray voltage adjust to 2.4 kV. The proteome discovery software suite (Thermo Fisher Scientific v2.1) was used to query MS/MS spectral results in the SwissProt human database within Uniprot (www.Uniprot.org). At the level of proteins, we chose a filter of a 1% false discovery rate (FDR) to ensure that each protein contained at least one unique peptide. Significance was determined based on a fold change > 1.5 and *P* value < 0.05.

### Western blotting

Urinary exosome samples were lysed by RIPA lysis buffer containing phenyl methane sulfonyl fluoride (PMSF) for 30 minutes. A total of 8 ug urinary proteins were loaded on 10 ~ 20% sodium dodecyl sulfate-polyacrylamide gel. After that, the electrophoresis was performed under 60 ~ 120V, and then the membrane was transferred using a rapid transfer system for a duration of 7 to 12 minutes (Bio-Rad, TURBO, USA). A solution containing 5% skim milk and 1xTBST was prepared and placed on a shaker at a temperature of 26 °C for 2 hours. The blots were properly cut prior to hybridization with antibodies during blotting. Then, Anti-CD9 (Abcam, ab236630), Anti-CD63 (Proteintech, Cat No. 67605-1-Ig), Anti-TSG101 (Proteintech, Cat No. 28283-1-AP), Anti-Calnexin (Proteintech, Cat No. 10427-2-AP), Anti-EGFR (Proteintech, Cat No. 66455-1-Ig), and Anti-PAK6 (Proteintech, Cat No. 13539-1-AP) were diluted at a ratio of 1:1000 ~ 3000, respectively, and followed by incubation in a refrigerator at a temperature of 4 °C. After three times washing the membrane with 1xTBST in the next day, secondary antibody (HRP conjugated anti-Rabbit IgG, Lot:158,560; HRP conjugated anti-Mouse IgG, Lot:150,976) was added at a ratio of 1:3000 for 1.5 hours. Lastly, the membrane was rewashed, and chemiluminescent ECL solution was added (Bio-Rad, cat. #170–5061) for exposure. The representative raw bands of Western Blot were shown in Supplementary materials 2.

### ELISA

Lysis of 72 urinary exosome samples for at least 30 minutes as previously mentioned. The concentrations of PAK6 and EGFR were measured by *ELISA* kit (Shanghai Enzyme-linked Biotechnology Co., Ltd; lot:202,302, Catalog number: YJ008742 and YJ022112). Based on the directions, 50 µl of each sample was added to well and incubated at a temperature of 37 °C for 30 minutes. The plate was washed five times, and then the HRP-Conjugate reagent was added and incubated for 30 minutes at a temperature of 37 °C. Then, 50 µl chromogen solution A and 50 µl chromogen solution B was added and incubated at a temperature of 37 ℃ for 10 minutes. Finally, adding a total of 50 µl of stop solution to each well for subsequent absorbance tests. Each urinary exosome sample was tested three times. The optical density (OD) value was calculated at 450 nm (Multiskan FC, Thermo Fisher Scientific, USA), and further calculated the concentrations of target protein based on the standard curve.

### Statistical analysis

Mean ± standard deviation was used to express all quantitative data for the normal distribution data, while median with interquartile range was used to express the non-normal distribution data. Categorized variables were presented by numbers (proportions). For continuous variables, the student’s t test or nonparametric tests were used to compared. For categorical variables, Pearson x^2^ test was used to compared two groups. The correlations between serum biomarkers and urinary exosome protein concentrations were analyzed using Pearson’s or Spearman’s correlation analysis. The statistical data were analyzed using SPSS 24.0 software (SPSS Inc., Chicago, IL, USA). Statistical significance was determined by a *P* value less than 0.05.

## Results

### The clinical characteristics of participants

In this study, 24 healthy subjects, 24 patients with T2DM, and 24 patients with DN were randomly selected as the discovery phase and further performed LC-MS/MS analysis. Other participants were divided into the validation phase (n = 72). There were no significant differences between gender and age in the discovery and validation phases. Patients were diagnosed with DN had a higher level of SBP, FBG, HbA1c, and serum Cr than patients were diagnosed with T2DM (All *P* < 0.05). Patients with DN also had higher proportions of hypertension and diabetic retinopathy (All *P* < 0.01). Besides, a larger proportions of patients with DN had chosen insulin combined with hypoglycaemic agents as a treatment strategy (*P* = 0.003). Nevertheless, eGFR and serum ALB levels were lower in patients with DN compared with patients with T2DM (All *P* < 0.01). There were no statistically significant differences in other clinical characteristics. The clinical information of all participants was shown in Table 1.

**Table 1:** Characteristics of healthy controls and patients

Variables	HC (n = 48)	T2DM (n = 48)	DN (n = 48)	*P* value
Age, years	60 (50.25 ~ 65.0)	58.0 (49.5 ~ 63.0)	61(51.25 ~ 66.75)	0.196
Gender, Male, n (%)	28(58.33)	28 (58.33)	28(58.33)	1.000
Hypertension, yes, n (%)	5 (10.42)	20(41.67)	36(75.0)	0.002
CHD, yes, n (%)	3(6.25)	16(33.33)	14(29.17)	0.826
Smoking history				0.642
Yes, n (%)	5 (10.41)	11 (22.92)	14 (29.67)	
No, n (%)	43 (89.59)	37 (77.08)	34 (70.83)	
Drinking history				1.000
Yes, n (%)	7 (14.58)	5 (10.42)	6 (12.5)	
No, n (%)	41 (85.42)	43 (89.58)	42 (87.5)	
Diabetic retinopathy				< 0.001
Yes, n (%)	NA	2(4.17)	27(56.25)	
No, n (%)	NA	46(95.83)	21(47.35)	
Diabetic foot				1.000
Yes, n (%)	NA	0(0)	0(0)	
No, n (%)	NA	48(100)	48(100)	
Diabetic family history				0.301
Yes, n (%)	8 (16.67)	25(47.92)	31(64.58)	
No, n (%)	40 (83.33)	23(52.08)	17(35.42)	
DD, years				0.146
< 5 years	NA	10(21.83)	4(8.33)	
≥ 5 years	NA	38(79.17)	44(91.67)	
BMI, kg/m^2^	22.84 ± 2.75	25.47 ± 3.42	27.02 ± 4.95	0.397
SBP, mm Hg	119.96 ± 8.32	127.0(117.5 ~ 138.5)	138.5 ± 20.24	0.023
DBP, mm Hg	75 (70.0 ~ 78.75)	79.58 ± 9.67	80.0(76.5 ~ 87.75)	0.187
AST, U/L	19.19 ± 3.05	20.0 (15.0 ~ 25.75)	19.5(12.0 ~ 28.0)	0.602
ALT, U/L	18.52 ± 5.59	17.0(14.0 ~ 20.75)	16.0 (13.0 ~ 20.75)	0.607
ALB, g/L	44.00 ± 2.32	40.16 ± 2.78	36.78 ± 2.79	< 0.001
FPG, mmol/L	5.09 ± 0.38	6.78 (6.02 ~ 8.33)	8.01 (6.66 ~ 11.16)	0.007
HbA1c, %	5.4 (5.2 ~ 5.5)	8.87 ± 1.90	9.41 ± 2.18	0.037
GA, %	9.67 ± 1.64	20.60 (17.25 ~ 28.10)	25.16 ± 7.31	0.101
Serum Cr, µmol/L	64.52 ± 11.57	66.0(51.25 ~ 69.75)	78.0 (63.0 ~ 91.5)	< 0.001
eGFR, mL/min/1.73m^2^	104.79 ± 9.72	100.5(95.25 ~ 108.75)	85.00 (63.25 ~ 100.5)	< 0.001
UACR, mg/g				< 0.001
<30	48 (100.00)	48(100.00)	0(0)	
≥ 30	0 (0)	0(0)	48(100.00)	
Treatment				0.003
Insulin + Drugs, n (%)	NA	10(21.83)	24(50.0)	
Drugs, n (%)	NA	38(79.17)	24(50.0)	

**Note**: Values are presented as mean ± standard deviation, number (%), or median (interquartile range); *P* value: T2DM vs. DN

### Characterization of exosomes derived from urine samples

**Fig. 2:**
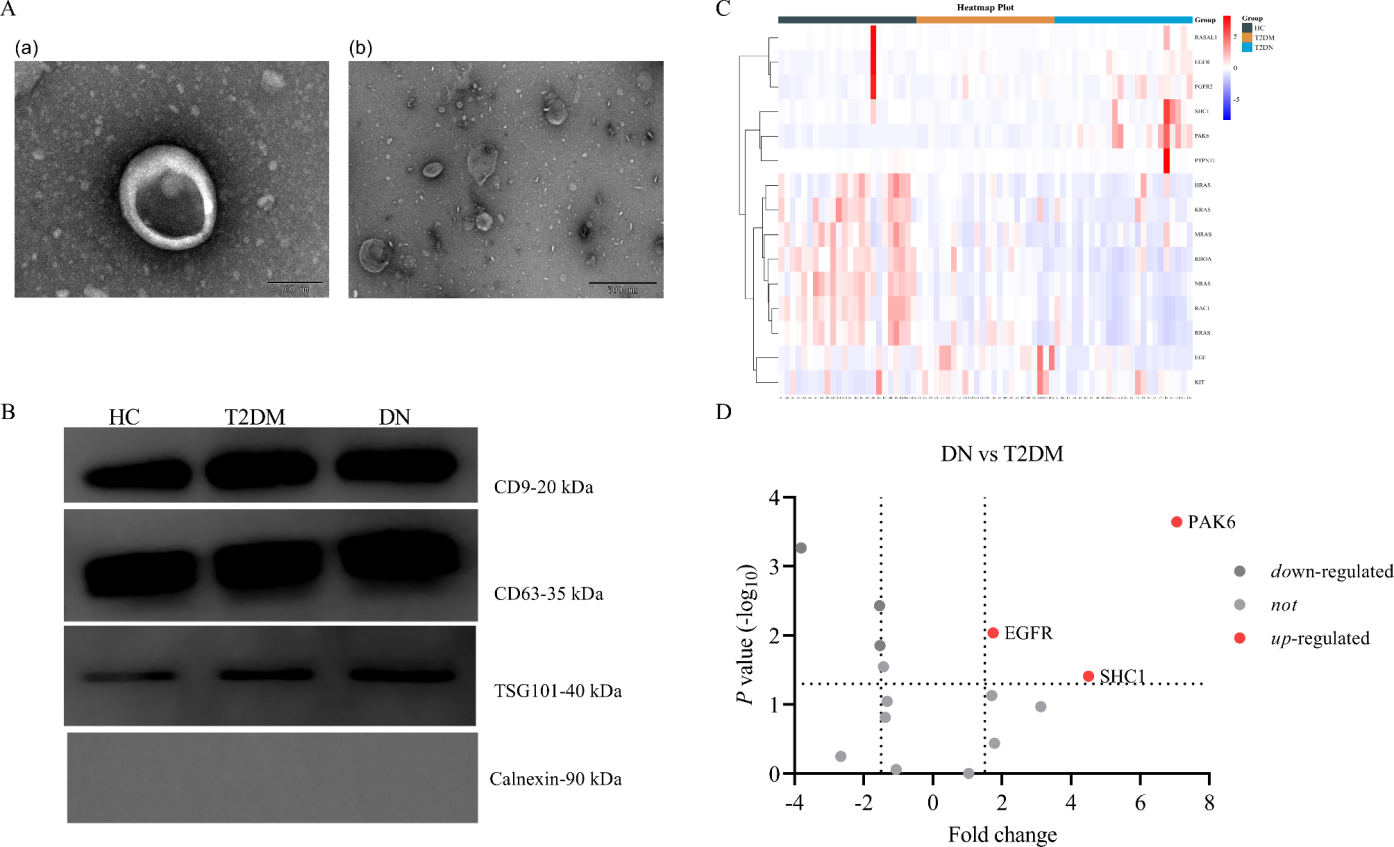
**Identification of urinary exosomes and proteomic analysis of proteins related to regulating actin cytoskeleton**. (**A**) Typical TEM images of urinary exosomes (scale bars; a = 100 nm, b = 500 nm). (**B**) Western blot images of CD9, CD63, TSG101, and Calnexin (The blots were properly cut prior to hybridisation with antibodies during blotting). (**C**) Hierarchical clustering heatmap analysis of 15 urinary proteins in the three groups. (**D**) Volcano analysis of urinary exosome proteins between T2DM, and DN groups. The abscissa is represented by fold change and the ordinate is represented by -log_10_ (*P* value)

The present study extracted urinary exosomes from the urine samples, and then measured TEM to observe the morphology of exosomes. The representative double-membrane oval shape was observed (Fig. 2A: a, 100 nm; b, 500 nm). The mean size of urinary exosomes was 122.9 nm using NTA analysis (Supplementary Materials 1). Four exosome markers, including CD9, CD63, TSG101, and Calnexin were measured by Western blot (Fig. 2B). The results of western blot demonstrated that the exosomes isolated from urine samples clearly expressed CD9, CD63, and TSG101. The results of Calnexin showed that the purity of exosomes is high, and subsequent experiments can be carried out (Fig. 2B).

### Regulation of actin cytoskeleton proteins in urinary exosomes

Fifteen urinary exosome proteins related to regulation of actin cytoskeleton were detected, and the hierarchical clustering heatmap analysis was showed in Fig. 2C. A fold change > 1.5 and *P* value < 0.05 was considered significantly different expression and the volcano plot between T2DM and DN groups was presented in Fig. 2D. Compared with patients with T2DM, the abundance of three urinary exosome proteins was increased, including Serine/threonine-protein kinase PAK6 (PAK6), Epidermal growth factor receptor (EGFR), and SHC-transforming protein 1(SHC1).

### Bio-functional analysis of 15 urinary exosome proteins

**Fig. 3:**
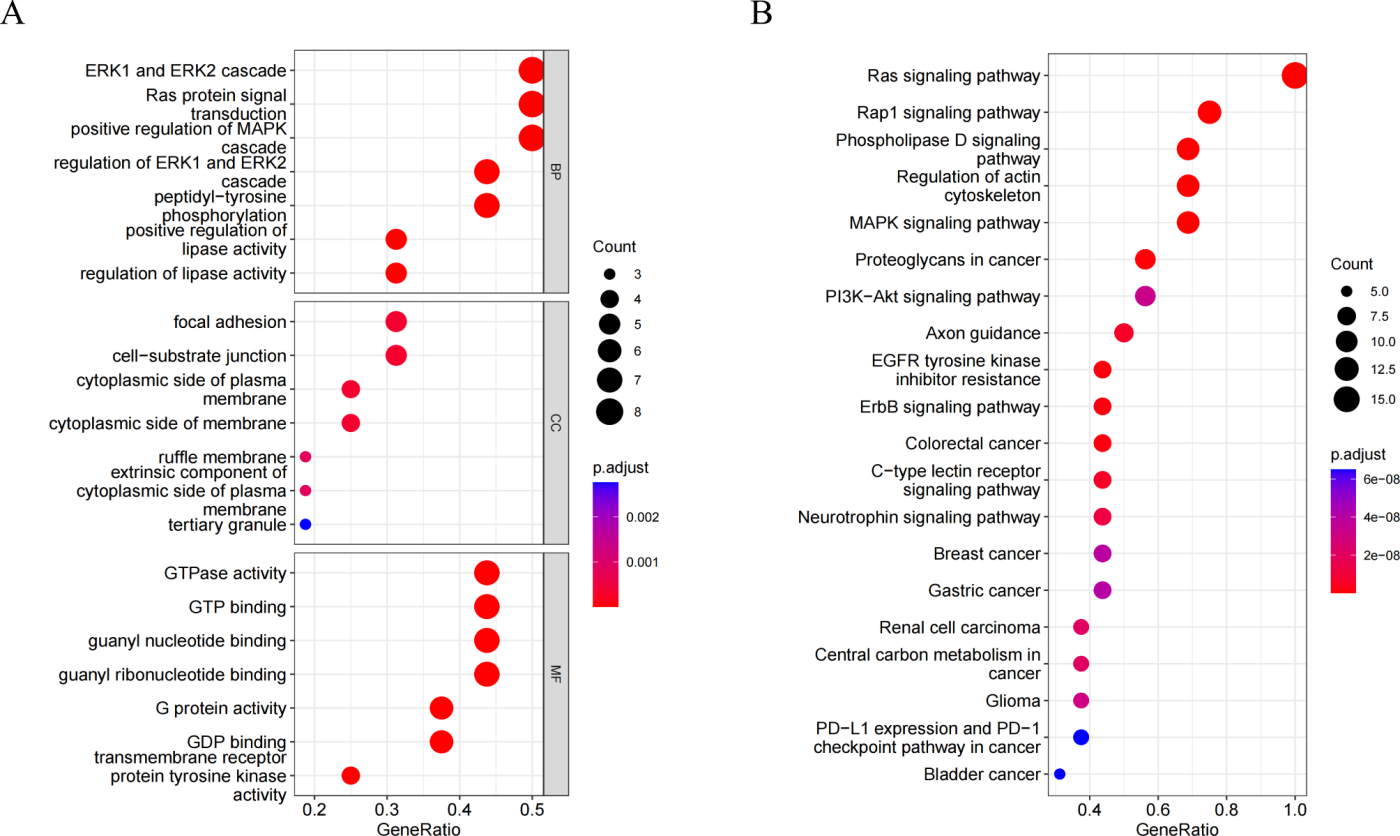
**Bioinformatics analysis of 15 urinary exosome proteins**. (**A**) GO enrichment analysis of 15 proteins. The ordinates represent GO functional categories: biological process (BP), molecular function (MF), and cellular component (CC). The horizontal axis represents the proportion of protein, the size of the dot represents the number of genes, and the color represents the size of the *P* value. (**B**) KEGG enrichment analysis of 15 proteins. The vertical axis represents the significantly enriched KEGG pathways, the horizontal axis represents the proportion of proteins, the size of the dots represents the number of genes, and the color represents the size of the *P* value

Biological function analysis demonstrated that these proteins were well associated with ERK1 and ERK2 cascade, Ras protein signal transduction, positive regulation of MAPK cascade, and regulation of ERK1 and ERK2 cascade. The majority of the cellular components were located on the focal adhesion, and their molecular functions were mostly related to GTPase activity (Fig. 3A). The Ras signaling pathway, Rap1 signaling pathway, Phospholipase D signaling pathway, and Regulation of actin cytoskeleton were the most abundant functions through KEGG pathway analysis (Fig. 3B).

### Differences in the expression level of urinary exosome proteins

**Fig. 4:**
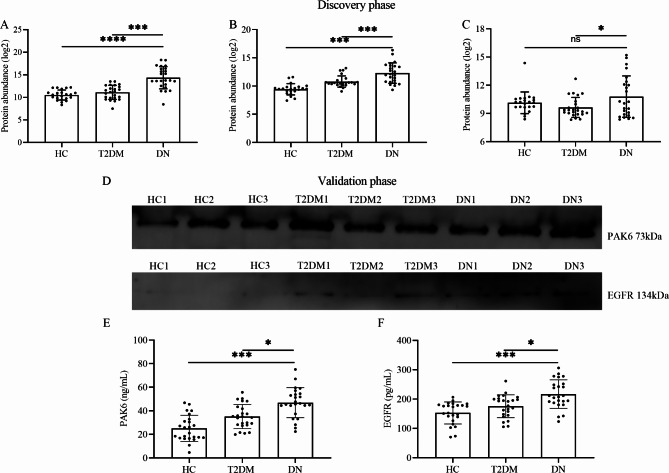
**Differences in the expression of three urinary exosome proteins**. Discovery phase: (**A, B, and C**) The Difference in the expression of PAK6, EGFR, and SHC1 among healthy control, T2DM, and DN groups. Validation phase: (**D**) Western blot images of urinary exosome PAK6 and EGFR proteins (The blots were properly cut prior to hybridisation with antibodies during blotting.). (**E and F**) The Difference in the expression of PAK6 and EGFR by ELISA analysis. (ns: no significance; *:*P* < 0.05; **P < 0.01; ***: *P* < 0.001)

In the discovery phase, patients with DN had higher levels of three urinary exosome proteins than patients with T2DM (All *P* < 0.05, Fig. 4A, B, and C). Furthermore, the expression levels of PAK6 and EGFR were significantly different in healthy people, excluding the SHC1 (*P* = 0.1382, Fig. 4C).

To further confirm that the differences were observed, the results of Western blot showed that the increasing trends of the urinary exosome proteins PAK6 and EGFR were found in patients with DN (Fig. 4D). Meanwhile, 72 samples were tested by ELISA analysis. The results demonstrated that patients were diagnosed with DN had highest concentrations of urinary exosome proteins PAK6 and EGFR among participants (All *P* < 0.05; Fig. 4E and F).

### Correlation analysis between the expression levels of urinary exosome proteins and serum Cr and eGFR levels

**Fig. 5:**
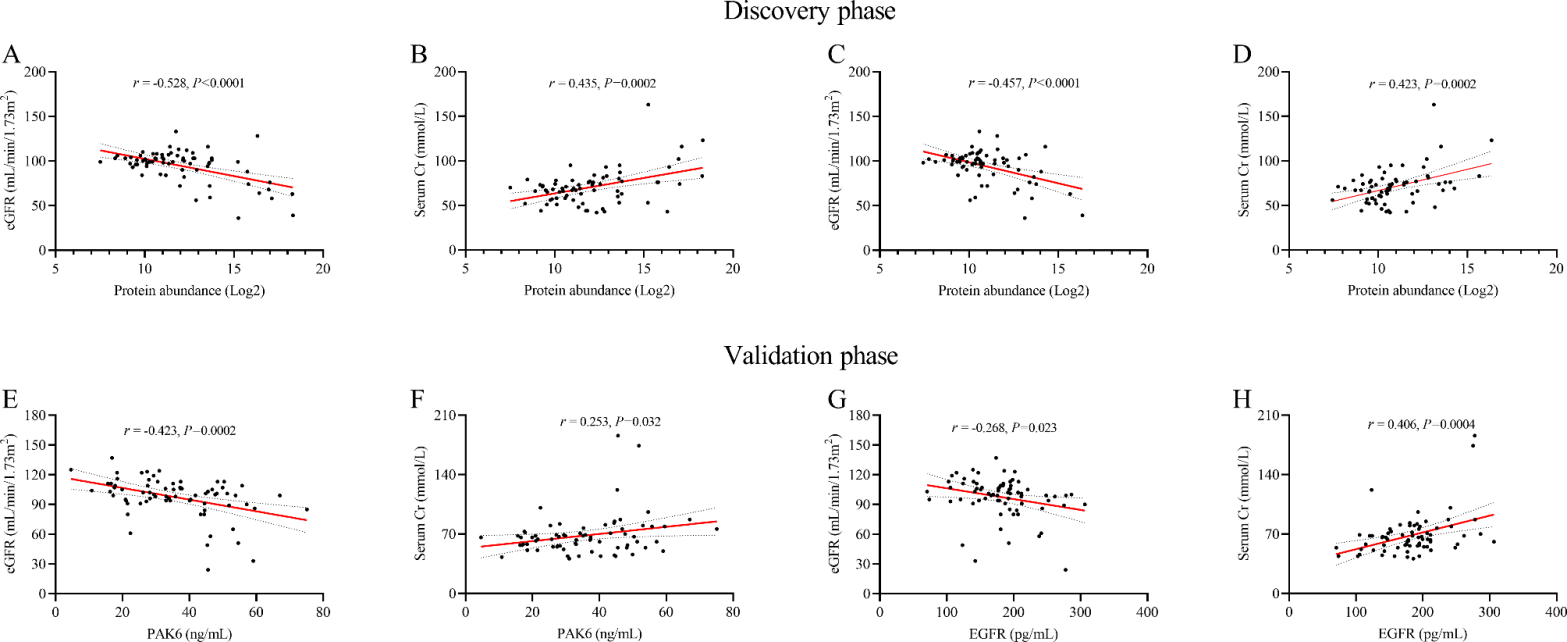
**The correlation analysis between urinary exosome proteins and serum renal function biomarkers**. (**A, B, C and D**) The correlation analysis between the expression level of PAK6 and EGFR and eGFR and serum Cr levels in the discovery phase. (**E, F, G and H**) The correlation analysis between the expression level of PAK6 and EGFR and eGFR and serum Cr levels in the validation phase. (ns: no significance; *:*P* < 0.05; **P < 0.01; ***: *P* < 0.001)

In the discovery phase, the correlation between the abundance of upregulated urinary exosome proteins and the level of serum Cr and eGFR were analyzed. The results demonstrated that the abundance of PAK6 was negatively correlated with eGFR levels (*r*=-0.528, *P* < 0.0001, Fig. 5A) and positively correlated with serum Cr levels (*r* = 0.435, *P* = 0.0002, Fig. 5B). The abundance of EGFR was negatively correlated with eGFR levels (*r*=-0.457, *P* < 0.0001, Fig. 5C) and also positively correlated with serum Cr levels (*r* = 0.423, *P* = 0.0002, Fig. 5D). Although the abundance of SHC1 was negatively correlated with eGFR levels (*r*=-0.101, *P* = 0.022, Supplementary Materials 3), no correlation was found in serum Cr levels (*P* = 0.173, Supplementary Materials 3).

In the validation phase, the negative correlation was observed between PAK6 concentrations and eGFR levels (*r*=-0.423, *P* = 0.0002, Fig. 5E). Besides, positive correlation was found between PAK6 concentrations and serum Cr levels (*r* = 0.253, *P* = 0.032, Fig. 5F). The EGFR concentrations was negatively correlated with eGFR levels (*r*=-0.268, *P* = 0.023, Fig. 5G) and positively correlated with the level of serum Cr (*r* = 0.406, *P* = 0.0004, Fig. 5H).

### Significance of PAK6 and EGFR in diagnosing of DN

**Fig. 6:**
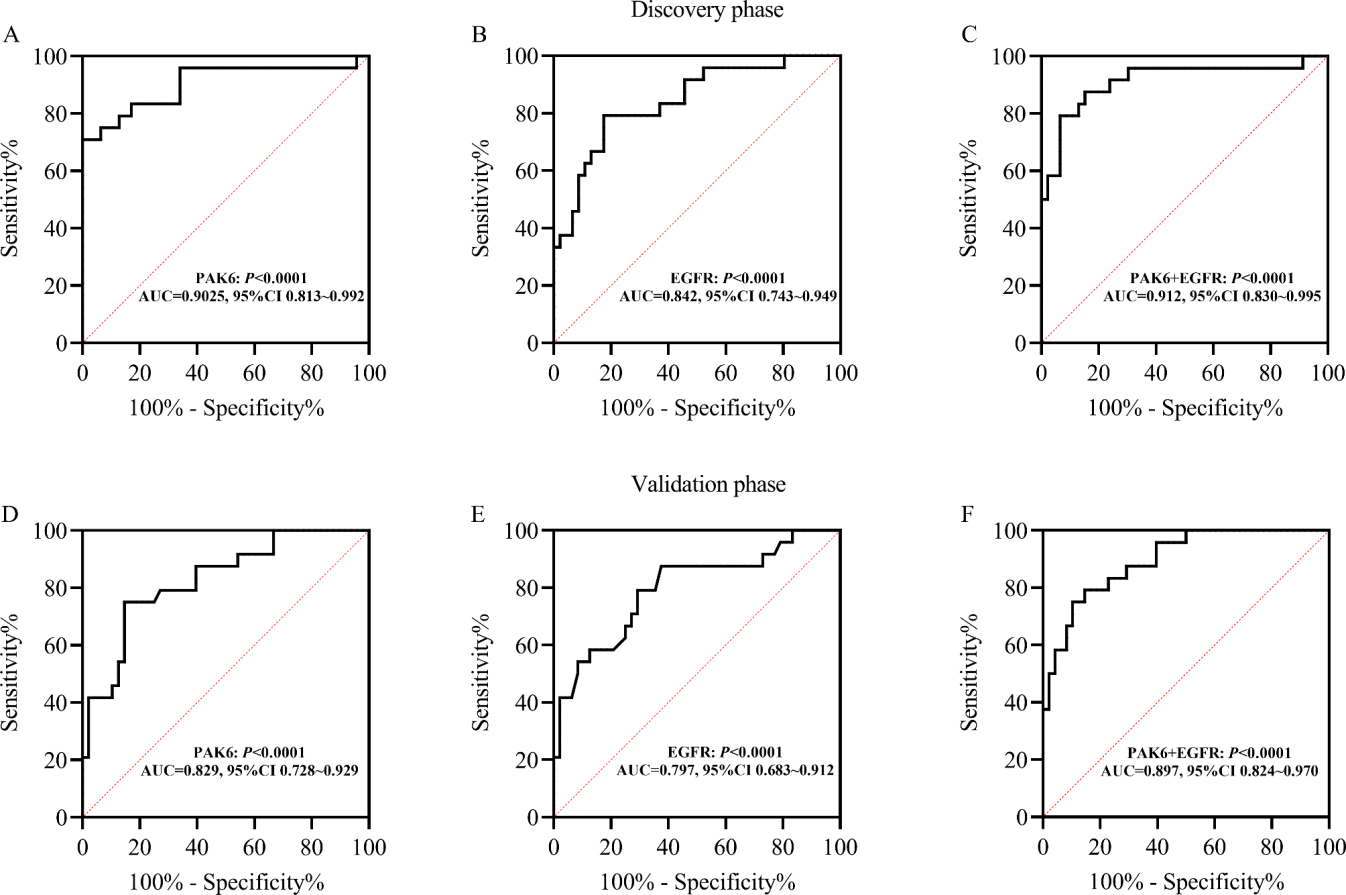
**ROC curve analysis of urinary exosome proteins PAK6 and EGFR**. Discovery phase: (**A**) PAK6, (**B**) EGFR, and (**C**) the combination of PAK6 and EGFR. Validation phase: (**D**) PAK6, (**E**) EGFR, and (**F**) the combination of PAK6 and EGFR. AUC: area under curve; 95% CI: 95% confidence interval

In the discovery phase, the predicting value of urinary exosome proteins PAK6 was shown in Fig. 6A-C. For the urinary exosome protein PAK6: the area under the curve (AUC) was 0.903 (95% CI, 0.813 ~ 0.992, *P* < 0.0001), and the AUC of EGFR was 0.842 (95%CI, 0.743 ~ 0.949, *P* < 0.0001). When two urinary exosome proteins were used in combination, the AUC was 0.912 (95% CI, 0.830 ~ 0.995, *P* < 0.0001).

In the validation phase, the predicting value of PAK6 and EGFR was shown in Fig. 6D-F. For the urinary exosome protein PAK6: The area under the curve (AUC) was 0.829 (95% CI, 0.728 ~ 0.929, *P* < 0.0001), and the AUC of EGFR was 0.797 (95%CI, 0.683 ~ 0.912, *P* < 0.0001). When two urinary exosome biomarkers were used in combination, the AUC was 0.897 (95% CI, 0.824 ~ 0.970, *P* < 0.0001).

## Discussion

Podocytes injury has been considered an important early event and the most powerful predictor for the development and progression of DN [18, 19]. With the understanding of DN, alterations in the actin cytoskeleton are closely associated with podocyte injury [4, 20]. In the present study, we identified 15 proteins related to regulating actin cytoskeleton, and further selected 3 upregulated proteins, including PAK6, EGFR, and SHC1. Ras protein signal transduction, GTPase activity, focal adhesion, and regulation of actin cytoskeleton were the most abundant enrich functions by GO and KEGG analysis, which further supports that the actin cytoskeleton plays an essential role in maintaining podocyte structure. Among the three groups, the urinary exosome proteins PAK6 and EGFR had the highest expression level and SHC1 was not. The eGFR levels were considered an important indicator for clinical diagnosis of DN and serum Cr levels also served as a powerful biomarker to evaluate renal function. We further explored the correlation between the expression level of urinary exosome proteins and these two markers in the discovery and validation phases. The results showed that the expression level of PAK6 and EGFR was positively correlated with serum Cr levels and negatively correlated with eGFR levels. This study further evaluated the value of PAK6 and EGFR for predicting DN. PAK6 and EGFR had better performance for diagnosing in two cohorts by ROC curve analysis. These results showed that elevated urinary exosome proteins PAK6 and EGFR may be promising, noninvasive, and powerful diagnostic biomarkers for patients with DN. What follows is a description of the possible reasons why PAK6 and EGFR play a key diagnostic role in DN.

PAK6 is a key member of a family of class II p21-stimulated serine/threonine protein kinases, which contain a carboxyl-terminal kinase domain and an amino-terminal Cdc43/Rac interactive binding domain [21]. PAK6 protein was involved in a variety of cellular functions, including gene transcription, cytoskeleton formation, cell motility, drug resistance, and cell apoptosis [22, 23]. PAK6 has been well established as an important regulated protein in different types of cancer such as gastric cancer [24], hepatocellular carcinoma [25], cervical cancer [26], prostate cancer [22]. Moreover, Lin and colleagues demonstrated that PAK6 was associated with the chemosensitivity of anti-cancer drugs for chronic myeloid leukemia [27]. In our literature investigations, there were very few relevant studies on the relationship between PAK6 and DN. This reminded us to further expand the clinical samples to validate the reliability of PAK6 in monitoring DN, as well as to explore more deeply its potential mechanisms of kidney damage in vivo and in vitro.

EGFR is a member of receptor family that contains tyrosine kinase activity and consists of four members: EGFR (ErbB1), ErbB2, ErbB3, and ErbB4. Activation of these receptors can occur through several ligands, such as EGF, transforming growth factor-α, amphiregulin, heparin-binding EGF-like growth factor and et al [28, 29]. EGFR is widely expressed in glomeruli and proximal tubules and the role of EGFR involved in the pathogenesis of DN has been extensively studied [30, 31]. In animal models of diabetes and cultured cells treated with high glucose, the level of phosphorylation of renal EGFR was significantly increased [32–35]. EGFR activation by high glucose contributes to multicellular dysfunction, which initiates and accelerates kidney injury. However, Inhibition of EGFR could slow the progression of DN, including improvement of proteinuria and morphological changes [36–38]. In a word, the pathogenesis of EGFR-mediated DN involves altered hemodynamic, metabolic disorders, inflammatory and immune responses, and kidney cellular dysfunction. In this study, a higher level of EGFR expression was observed in urinary exosomes and correlated with serum Cr and eGFR levels, suggesting that activation of EGFR may continuously exist in patients with DN and can be excreted out of the body through the urine. In conclusion, as well as PAK6, still needs more intensive studies to validate and reveal the mechanism of renal injury by the urinary exosome protein EGFR.

We were also aware of some limitations in our study. The sample size of the patients that enrolled in the study was not large and the results of single-center retrospective cohort studies were not generalizable to other populations. The predicting value of urinary exosome protein PAK6 and EGFR needs to be further evaluated and validated by expanding the sample size based on a multicenter cohort. Besides, our team has not explored which these urinary exosome proteins are related to, and more studies in vivo and in vitro are needed in the future.

## Conclusions

In the present study, we explored the expression differences of regulating actin cytoskeleton in urinary exosomes. Urinary exosome proteins PAK6 and EGFR correlated with serum Cr and eGFR levels. The upregulated urinary exosome proteins PAK6 and EGFR could serve as promising and novel biomarkers for diagnosing DN.

### Electronic supplementary material

Below is the link to the electronic supplementary material.

Supplementary Material 1

Supplementary Material 2

Supplementary Material 3

## Data Availability

The data in the current study could be available from the corresponding author on reasonable request.

## List of Abbreviations

DN: Diabetic nephropathy
DEPs: Differential expression proteins
T2DM: Type 2 diabetes mellitus
FBG: Fasting blood glucose
HbA1c: Glycosylated hemoglobin
eGFR: Estimated glomerular filtration rate
UACR: Urinary albumin/creatinine ratio
SBP: Systolic blood pressure
DBP: Diastolic blood pressure
CHD: Coronary heart disease
DD: Diabetes duration
BMI: Body mass index
SBP: Systolic blood pressure
DBP: Diastolic blood pressure
AST: Aspartate aminotransferase
ALT: Alanine aminotransferase
ALB: Albumin
GA: Glycated albumin
Cr: Creatinine
FDR: False discovery rate
GO: Gene Ontology
KEGG: Kyoto Encyclopedia of Genes and Genomes analyses
ROC: Receiver operating characteristic curve
PAK6: Serine/threonine-protein kinase PAK6
EGFR: Epidermal growth factor receptor
SHC1: SHC-transforming protein 1
AUC: Area under curve
