# Supplementary material for: Urinary exosome proteins PAK6 and EGFR as noninvasive diagnostic biomarkers of diabetic nephropathy
Source: BMC Nephrol. 2023 Oct 3;24:291. doi: 10.1186/s12882-023-03343-7 (PMC10548700; doi:10.1186/s12882-023-03343-7)
Supplement: Supplementary file 1 — Supplementary Material 1 [file 12882_2023_3343_MOESM1_ESM.pdf]

Electrophoresis & Brownian Motion
Video Analysis
Laser Scattering Microscopy

www.particle-metrix.de

Operator (Report): Administrator
Video Operator: Administrator

Sample Parameters
Sample Name: 251
Comment: 1:10, Sample Remarks0:
Sample Remarks1:
Sample Remarks2:
Electrolyte: pbs
Temperature: 23.53 °C sensed
pH 7.4 entered
Conductivity: 0.00 µS/cm entered

Result (sizes in nm)

	Number	Concentration	Volume
Median (X50)	106.2	106.2	202.3
Span	61.8	61.8	107.2

Concentration: 4.7E+8 Particles / mL
Dilution Factor: 50
Original Concentration: 2.3E+10 Particles / mL

Quality
Average Counted Particles per Frame: 1018
Number of Traced Particles: 368

Measurement Parameters
Cell S/N: NTA

Measurement Mode: Size Distribution 4 Cycles
11 Positions, 10 Removed for Analysis

Analysis Parameters
Max Area: 1000, Min Area: 5, Min Brightness: 20

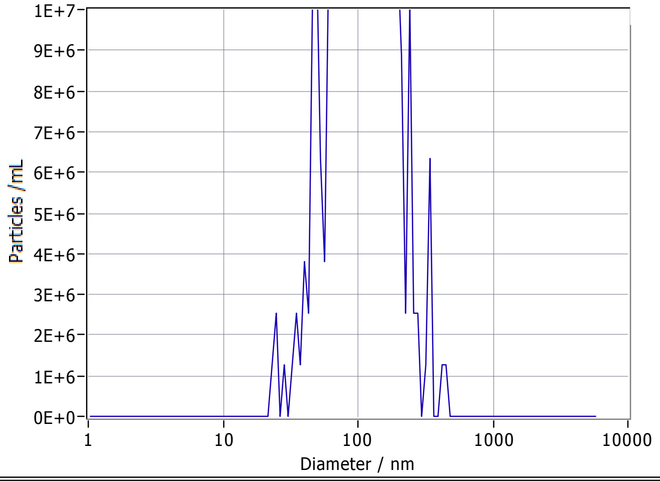

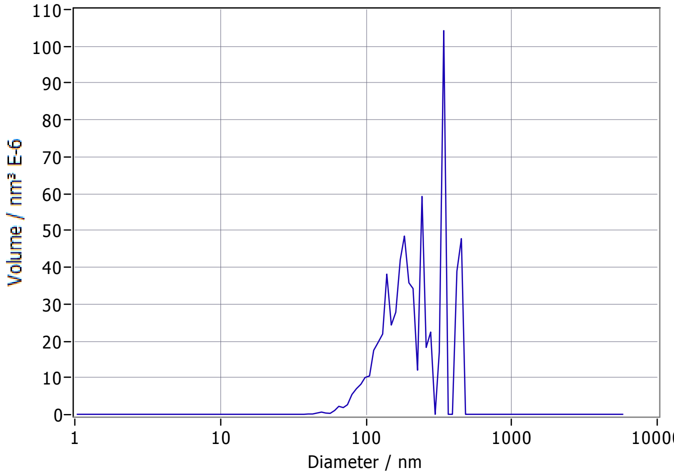

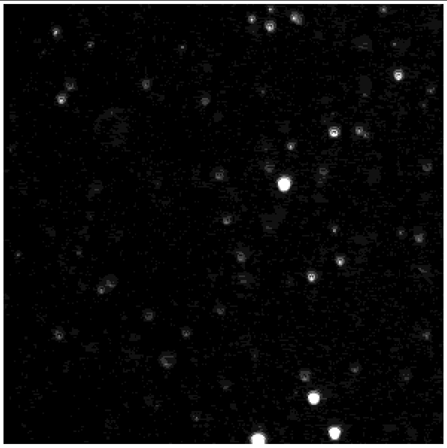

Peak Analysis (Concentration)

Diameter / nm	Particles/mL	FWHM / nm	Percentage
122.9	2.8E+7	128.1	91.7
342.4	4.1E+6	45.6	1.8
24.9	2.2E+6	2.3	0.8
474.9	1.2E+6	90.8	1.2
821.1	4.4E+5	194.0	0.7

X Values

	Number	Concentration	Volume
X10	55.2	55.2	113.1
X50	106.2	106.2	202.3
X90	185.2	185.2	405.6
Span	1.2	1.2	1.4
Mean	120.8	120.8	238.6
StdDev	61.8	61.8	107.2

Comment

(Signature)

Analyzed Video: E:\wen\221018\20221107_0002_251_size.avi

ZetaVIEW S/N 17-340, Software ZetaView 8.04.02, Camera 0.714 µm/px

Experiment: 2022-11-07 19:19, Report: 2022-11-07 19:46

Supplementary materials 1: The representative report of urinary exosomes concentrations by NTA.
