# Supplementary material for: Urinary exosome proteins PAK6 and EGFR as noninvasive diagnostic biomarkers of diabetic nephropathy
Source: BMC Nephrol. 2023 Oct 3;24:291. doi: 10.1186/s12882-023-03343-7 (PMC10548700; doi:10.1186/s12882-023-03343-7)
Supplement: Supplementary file 2 — Supplementary Material 2 [file 12882_2023_3343_MOESM2_ESM.pdf]

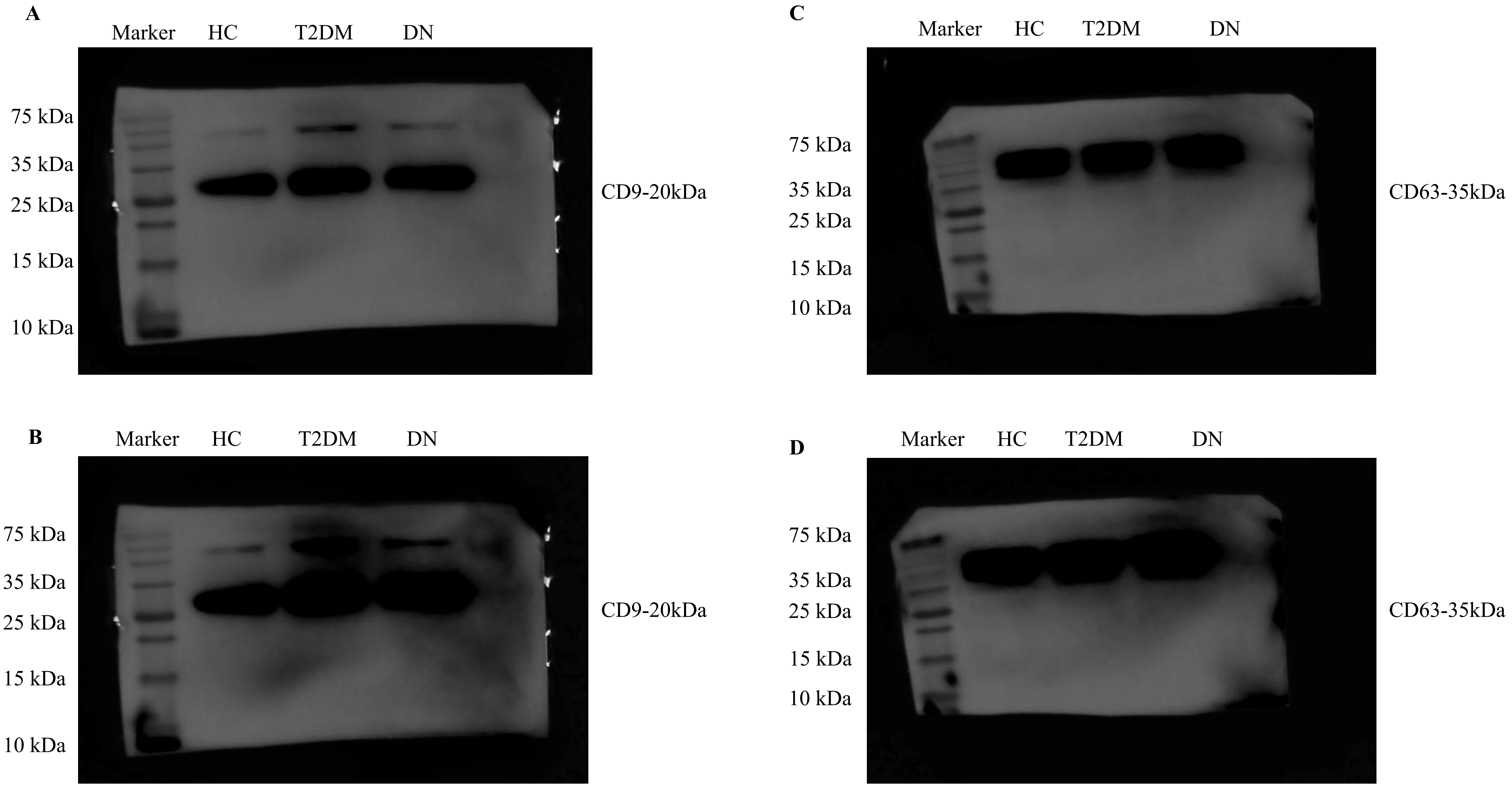

Supplementary materials 2 legends: A and B: The representative raw images of CD9 in different linear signal ranges. **C and D:** The representative raw images of CD63 in different linear signal ranges (The blots were properly cut prior to hybridization with antibodies during blotting).

E

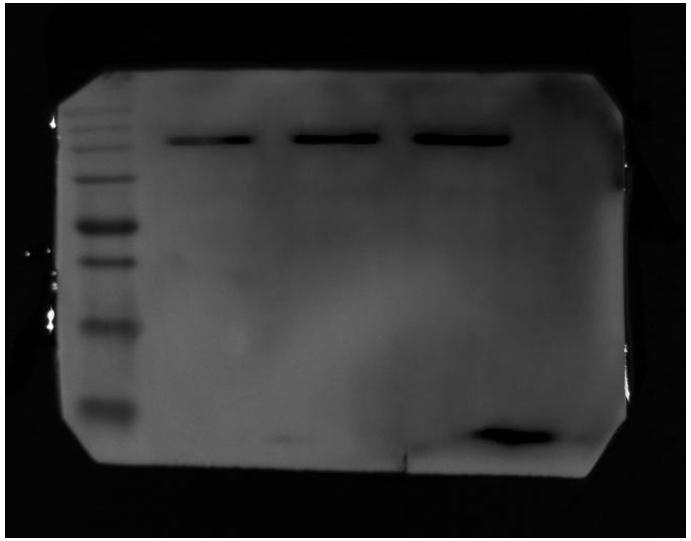

G

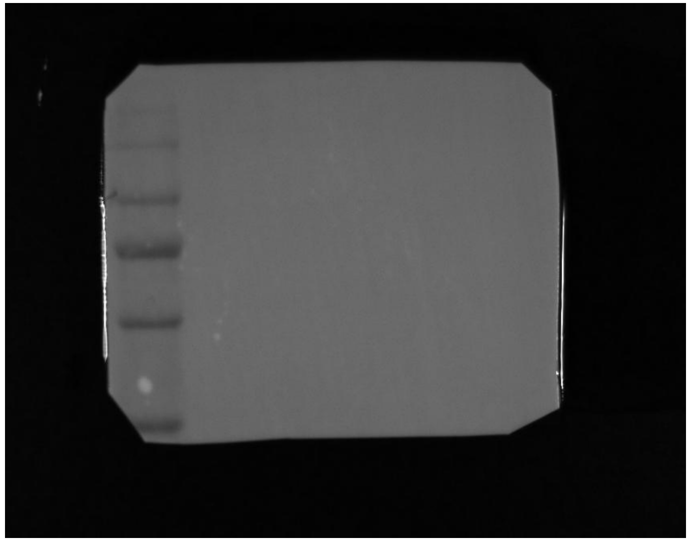

F

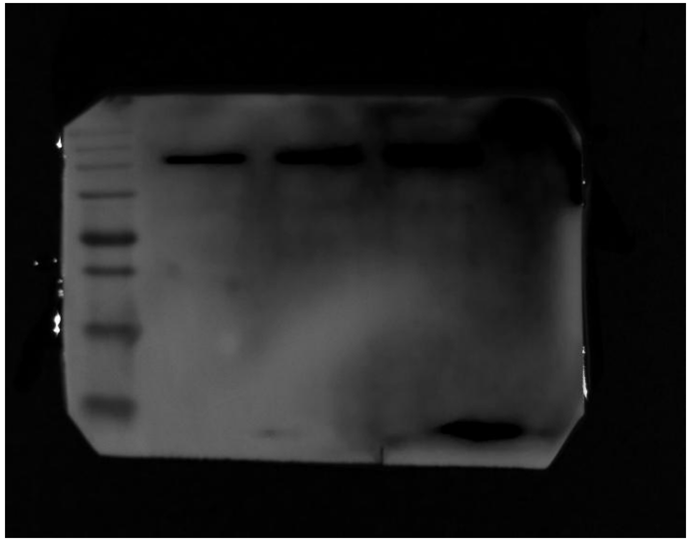

H

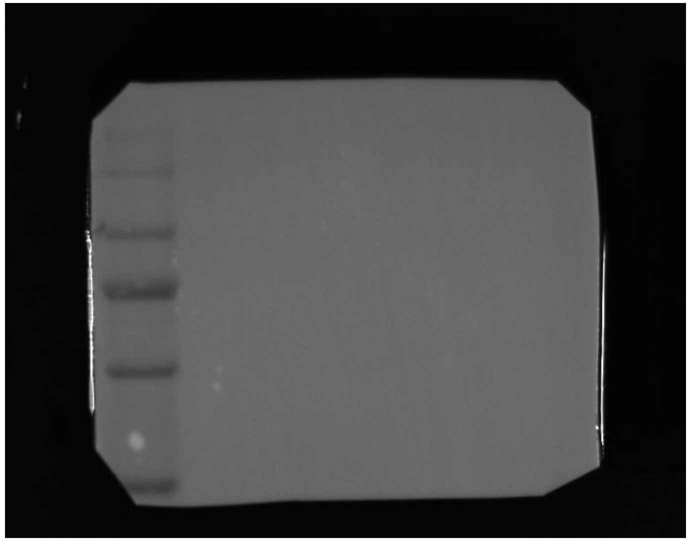

Supplementary materials 2 legends: E and F: The raw images of TSG101 in different linear signal ranges. **G and H:** The raw images of Calnexin in different linear signal ranges (The blots were properly cut prior to hybridization with antibodies during blotting).

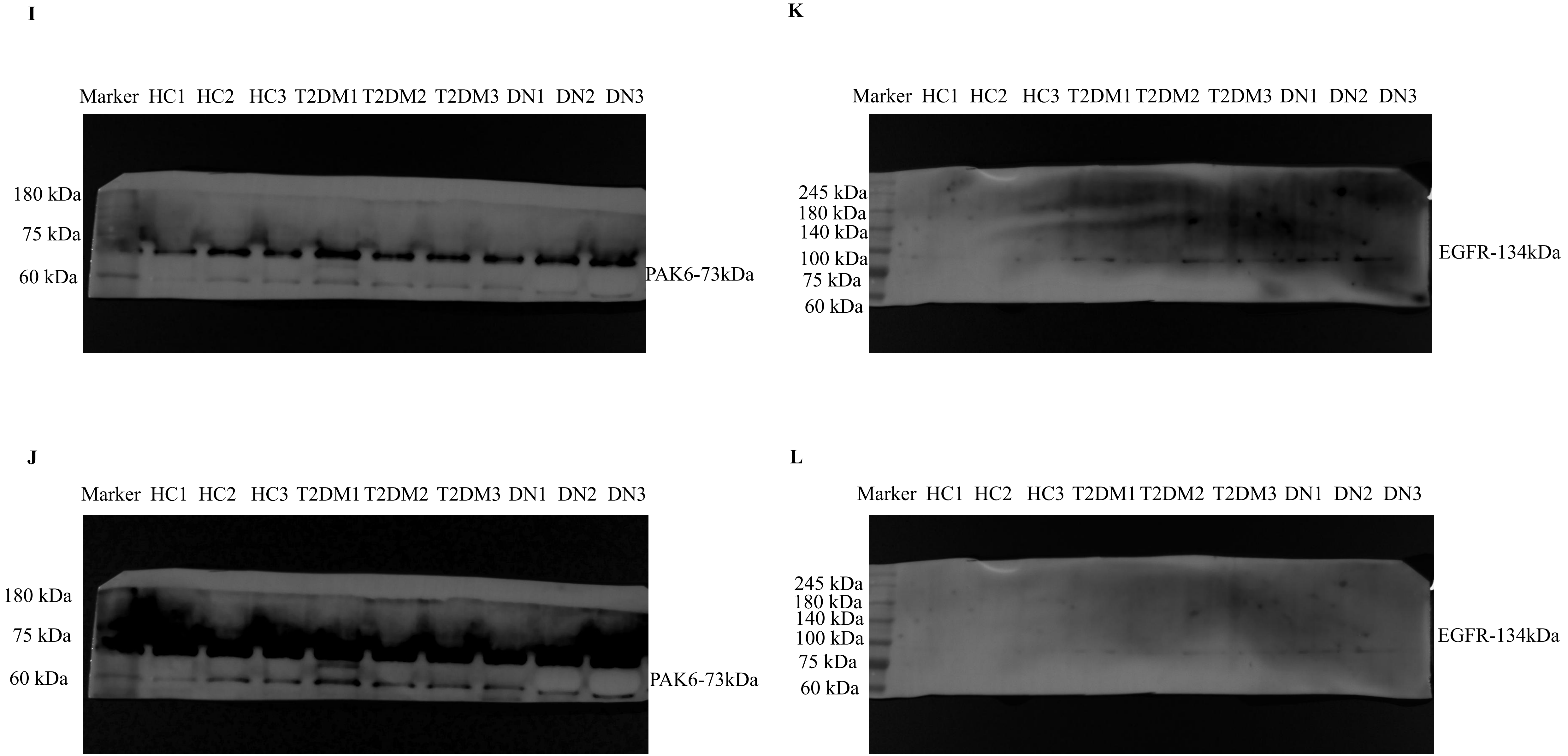

Supplementary materials 2 legends: I and J: The raw images of PAK6 in different linear signal ranges. **K and L:** The raw images of EGFR in different linear signal ranges (The blots were properly cut prior to hybridization with antibodies during blotting).
