# Supplementary material for: Urinary exosome proteins PAK6 and EGFR as noninvasive diagnostic biomarkers of diabetic nephropathy
Source: BMC Nephrol. 2023 Oct 3;24:291. doi: 10.1186/s12882-023-03343-7 (PMC10548700; doi:10.1186/s12882-023-03343-7)

Supplementary materials 3: The correlation analysis between the abundance of SHC1 and eGFR and serum Cr levels.

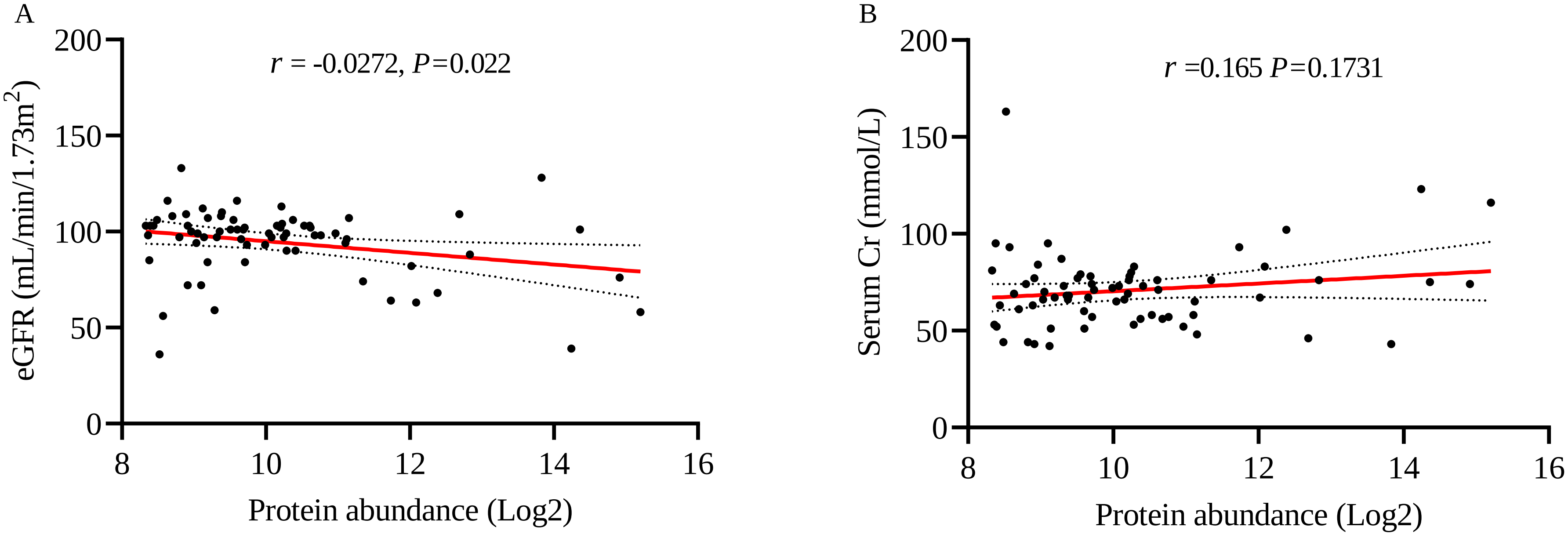

Supplement: Supplementary file 3 — Supplementary Material 3 [file 12882_2023_3343_MOESM3_ESM.pdf]
